# Prognostic nutrition index predicts short-term surgical complications in patients with rectal cancer after laparoscopic surgery

**DOI:** 10.3389/fsurg.2022.1000108

**Published:** 2022-10-25

**Authors:** Fengming Xu, Cong Meng, Zhengyang Yang, Haoze Li, Jiale Gao, Liting Sun, Xiao Zhang, Qi Wei, Guocong Wu, Hongwei Yao, Zhongtao Zhang

**Affiliations:** Department of General Surgery, Beijing Friendship Hospital, Capital Medical University, Beijing, China

**Keywords:** prognostic nutrition index, surgical complications, rectal cancer, laparoscopic surgery, short-term

## Abstract

**Purpose:**

Surgical complications following laparoscopic rectal cancer surgery remain a major clinical problem. The prognostic nutritional index (PNI) is reportedly associated with postoperative outcomes. We aimed to evaluate the correlation between PNI and short-term surgical complications in patients with rectal cancer after laparoscopic surgery.

**Methods:**

The prospective clinical data of 225 patients with rectal cancer receiving laparoscopic surgery between January 2021 and April 2022 were retrospectively analyzed. The cut-off values and diagnostic accuracy of PNI preoperatively and on postoperative day (POD) 1 were determined using receiver operating characteristic (ROC) curves. Univariate and multivariate analyses were performed to identify clinical characteristics and risk factors for surgical complications.

**Results:**

In total, 81 (36.0%) patients developed surgical complications. The optimal cut-off value for preoperative PNI was 40.15, and that for PNI on POD 1 was 35.28. The DeLong test found no statistically between–group difference in the area under the ROC curve (*P* = 0.598). Multivariate analysis identified that a preoperative PNI ≤40.15 [odds ratio (OR): 2.856, 95% confidence interval (CI): 1.287–6.341, *P* = 0.010] and PNI on POD 1 ≤35.28 (OR: 2.773, 95% CI: 1.533–5.016, *P* = 0.001) were independent risk factors for surgical complications. Patients with a preoperative PNI ≤40.15 or PNI on POD 1 ≤35.28 were more likely to have surgical complications after laparoscopic surgery for rectal cancer (61.1% vs. 31.2%, *P* = 0.001; 53.0% vs. 28.9%, *P* = 0.001).

**Conclusion:**

Preoperative and POD 1 PNI were independent predictors of short-term surgical complications after laparoscopic surgery for rectal cancer.

## Introduction

In 2020, there were reportedly more than 730,000 new cases of rectal cancer, with more than 330,000 deaths worldwide (1). The past few years see a gradual growing in the incidence of rectal cancer in China, posing a great threat to people's lives and health (2). With the continuous development of surgical technology, continuous progress of neoadjuvant therapy, and increasing demand of patients for anal preservation, the proportion of laparoscopic sphincter-sparing surgery for low and even ultralow rectal cancer is gradually increasing. Moreover, it also poses great challenges to the management of surgical complications.

Surgical complications may not only negatively impact short-term outcomes but also affect oncological outcomes (3, 4). Law et al. found that sepsis complications and anastomotic leakage were important factors for recurrence and speculated that changes in immune response related to sepsis might adversely affect the prognosis of tumors (5). Therefore, it is necessary to identify patients with poor postoperative outcomes early, because assessment and adjustment of modifiable risk factors of patients can serve as a potential window of opportunity to optimize postoperative outcome. It may be an effective method to prevent postoperative complications and improve overall survival by evaluating patients' nutritional and immunological conditions. It has been proposed that, with the aid of the Prognostic Nutrition Index (PNI), the nutritional status and the risk of postoperative complications in patients with gastrointestinal malignancies can be evaluated, which was calculated by methods of serum albumin concentration and peripheral blood lymphocyte count (6). Previous studies have confirmed that PNI is a useful predictor of postoperative complications and prognosis in patients with colorectal cancer (7–10). However, only a few studies and subgroup analyses of the surgical complications of rectal cancer exist.

Postoperative complications differ between rectal and colon cancers in that surgical complications are more common in rectal cancer patients (11). In addition, there are differences in treatment, including neoadjuvant treatment of locally advanced rectal cancer (12), the application of transanal endoscopic resection of mid-low rectal cancer (13), and defunctioning ileostomy to protect the low anastomosis. However, current studies on PNI in rectal cancer are insufficient. PNI is associated with the permanent stoma rate after anterior resection and defunctioning stoma (14), postoperative complications of locally recurrent rectal cancer (15), and postoperative complications of early rectal cancer (16). However, regarding the connection between PNI and postoperative surgical complications after laparoscopic surgery for rectal cancer, seldom reports are published. Hence, aiming at exploring the relationship between PNI and postoperative surgical complications after laparoscopic surgery for rectal cancer, this study was developed.

## Methods

### Patient selection

A retrospective analysis was carried out on the prospectively collected data of 225 consecutive patients who suffered from rectal cancer and have received laparoscopic surgery at Beijing Friendship Hospital, Affiliated with Capital Medical University between January 2021 and April 2022 were analyzed. The inclusion criteria were as follows: (1) 18–80 years of age; (2) colonoscopic diagnosis of the lower edge of the tumor within 15cm of the anal verge; (3) preoperative pathologically confirmed rectal adenocarcinoma; and (4) laparoscopic sphincter-sparing surgery and abdominal-perineal resection (APR) following total mesorectal excision (TME) or partial mesorectal excision principles. Exclusion criteria were as follows: (1) open surgery; (2) previous history of rectal surgery; (3) presence of intestinal obstruction, perforation, bleeding, or other emergency operations; (4) treatment with local excision (i.e., endoscopic, anorectal, or transanal endoscopic microsurgery approach); (5) presence of other primary malignancies during the same period; (6) histology other than adenocarcinoma; (7) presence of blood or immune system diseases; (8) presence of infection and non-cancer inflammatory diseases; and (9) parenteral nutrition support before surgery. All patients underwent colonoscopy and tumor biopsy before surgery. Except for those with contraindications, all patients underwent thoracoabdominal computed tomography (CT) and pelvic magnetic resonance imaging (MRI) to evaluate clinical staging after the histological diagnosis of rectal adenocarcinoma. This study gained approval from the Institutional Research and Ethics Committee.

### Data collection

The followings are included in the collected data: baseline characteristics, laboratory tests, and intraoperative indices. Baseline characteristics included age, sex, smoking, body mass index (BMI), comorbidities (hypertension, diabetes mellitus, and other diseases: respiratory diseases, cardiovascular, and cerebrovascular diseases), American Association of Anesthesiologists (ASA) score, preoperative tumor staging, tumor location [conditional on the distance between the lower edge of the tumor and the anal verge; they were classified as mid–low (0–10 cm), or high (10.1–15 cm)], neoadjuvant therapy, and previous abdominal and pelvic surgeries. Laboratory tests included preoperative serum albumin, lymphocyte count, C-reactive protein (CRP), hemoglobin, and serum albumin concentration and total lymphocyte count on postoperative day (POD) 1 (serum samples were collected before postoperative nutritional support). Intraoperative indices included operation time and method [anterior resection (AR) or low anterior resection (LAR), transanal TME (taTME), and APR], anastomosis, stoma form (permanent colostomy and defunctioning ileostomy), and intraoperative bleeding. Tumor stage and pathological tumor response grade was assessed according to the 8th American Joint Committee on Cancer Classification. All patients underwent high inferior mesenteric artery ligation, and anastomosis of patients undergoing anastomotic reconstruction was tension-free. The Enhanced Recovery After Surgery program was applied in all patients underwent laparoscopic surgery for rectal cancer.

Data regarding blood tests before the operation and on POD 1 were collected, including the serum albumin level and peripheral blood lymphocyte count. PNI calculation formula was: 10 × serum albumin value (g/dl) + 0.005 × peripheral blood lymphocyte count (per mm^3^).

### Definition of surgical complications

According to previous studies, short-term surgical complications were defined and recorded as surgical related complications that occurred within 30 days after surgery, including anastomotic leakage, surgical site infection, intestinal obstruction, abdominal and pelvic abscess, hemorrhage (intra-abdominal bleeding and anastomotic bleeding), urinary retention, urinary tract infection, and stoma related complications (stoma infection, stoma prolapse, stoma outlet obstruction, and high stoma output) (17–20). Surgical complications were categorized as per the Clavien–Dindo classification (21)—grades I + II: minor surgical complications, grades III + IV: major surgical complications. In this study, no mortality was found within 30 days after surgery (grade V). Diagnosed from the definition proposed by the international rectal cancer research group, anastomotic leakage was confirmed (22). All diagnoses of anastomotic leakage were supported by CT scan.

### Statistical analyses

All statistical analyses were performed using the R language package (version 3.6.3) and SPSS (version 26.0; IBM, Armonk, NY, USA). Categorical data were expressed as counts and percentages and the variables analyzed were by Fisher's exact or *χ*^2^ test. Likewise, continuous data were expressed as mean ± standard deviation and the variables analyzed by two-tailed *t*-test. With the aid of a receiver operating characteristic (ROC) curve, the specificity and sensitivity of PNI can be evaluated in predicting postoperative surgical complications. The optimal cut-off value was determined according to the maximum Youden index (sensitivity + specificity − 1). The DeLong test was used to compare the area under the ROC curve (AUC) of the preoperative and postoperative PNI. By means of univariate analysis the risk factors for surgical complications were analyzed. The statistically significant variables in the univariate analysis (*P* < 0.05), analyzed as covariates, were incorporated in the multivariate analysis model to determine the independent risk factors for surgical complications after laparoscopic rectal cancer surgery. A two-tailed *P*-value <0.05 was considered statistically significant.

## Results

### Patient characteristics

The clinical characteristics of the patients are detailed in Table 1. A total of 225 patients with rectal cancer who underwent laparoscopic surgery were included in this study: 148 (65.8%) men and 77 (34.2%) women. Their mean age and BMI were 62.7 ± 10.0 years and 23.9 ± 3.8 kg/m^2^, respectively. There were 99 (44.0%) cases of hypertension, 52 (23.1%) cases of diabetes, and 38 (16.9%) cases of respiratory, cardiovascular, and cerebrovascular diseases. Middle and low rectal cancer accounted for most cases of rectal cancer (*n* = 173, 76.9%), and the mean distance from the lower edge of the tumor to the anal verge was 80.9 ± 34.0 mm. A total of 71 (31.6%) patients received neoadjuvant therapy. All patients underwent laparoscopic surgery, with a mean operation time of 205.3 ± 60.1 min. Conventional laparoscopic surgery included AR/LAR in 167 (74.2%) cases, APR in 16 (7.1%), and taTME in 42 (18.7%). Primary anastomosis was performed in 205 (91.1%) patients. The defunctioning ileostomy rate was 56.4% (127/205), and 20 (8.9%) patients underwent permanent colostomy. The overall incidence rate of short-term surgical complications was 36.0% (81/225). Based on the Clavien–Dindo classification, 71 (31.6%) patients suffer from minor complications (grade I + II), 10 (4.4%) had major complications (grade III + IV), and 16 (7.1%) were rehospitalized due to surgical complications.

**Table 1:** Demographic and clinical features of patients.

Characteristic	*n* = 225
Age (year)	62.7 ± 10.0
Gender (*n*)	
Male	148
Female	77
BMI (kg/m^2^)	23.9 ± 3.8
Smoking (*n*)	49
Comorbidities (*n*)	
Hypertension	99
Diabetes mellitus	52
Others^a^	38
ASA ≥3 (*n*)	48
Tumor location (*n*)	
Upper rectum	52
Middle rectum	111
Lower rectum	62
Distance from anal verge (mm)	80.9 ± 34.0
Preoperative T stage (*n*)	
T1	5
T2	39
T3	110
T4	71
Preoperative N stage (*n*)	
N0	86
N1	87
N2	52
Preoperative M stage (*n*)	
M0	211
M1	14
Previous abdominal surgery (*n*)	46
Neoadjuvant therapy (*n*)	71
Preoperative serum albumin (g/L)	37.8 ± 3.4
Preoperative hemoglobin (g/L)	127.9 ± 18.4
Preoperative CRP (g/L)	3.4 ± 7.0
Preoperative lymphocyte count (×10^9^/L)	1.5 ± 0.7
Preoperative PNI	45.1 ± 5.3
Serum albumin on POD 1 (g/L)	32.8 ± 3.2
PNI on POD 1	37.1 ± 4.6
Mode of surgical approach (*n*)	
Conventional laparoscopic surgery	183
taTME	42
Anastomosis (*n*)	205
Colostomy/ileostomy (*n*)	147
Operation time (min)	205.3 ± 60.1
Blood loss (ml)	77.7 ± 60.2
Pathological TRG (*n*)	
0	15
1	30
2	19
3	7
Postoperative complications within 30 days (Clavien–Dindo) (*n*)	81
Grades I and II	71
Grades III and IV	10
Postoperative stay (days)	8.2 ± 4.7
Readmission within 30 days (*n*)	16
Deaths within 30 days (*n*)	0

BMI, body mass index; ASA, American Society of Anesthesiologists; CRP, C-reactive protein; PNI, prognostic nutritional index; POD, postoperative day; taTME, transanal total mesorectal excision; TRG, tumor response grade.

^a^ Respiratory system, cardiovascular and cerebrovascular system diseases were included.

### Predictive value of preoperative and POD 1 PNI for surgical complications

**Figure 1:**
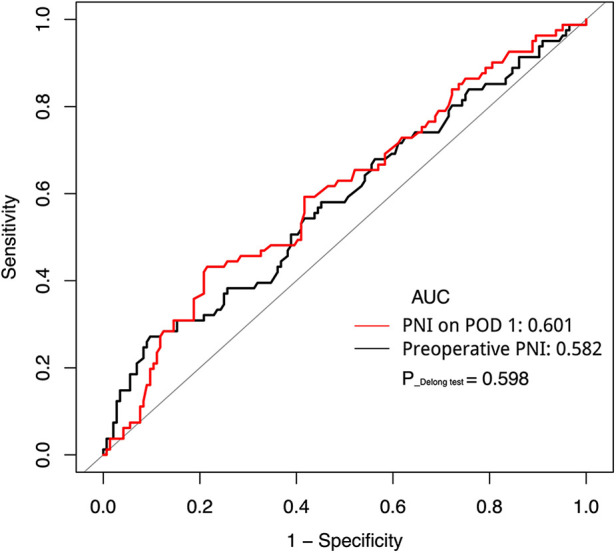
Receiver operating characteristic curve analysis of PNI preoperative and on POD 1 in predicting surgical complications. AUC, area under the curve; PNI, prognostic nutrition index; POD, postoperative day.

A ROC curve was adopted to compare the predictive accuracy of the preoperative PNI and PNI on POD 1. As shown in Figure 1, the AUC of the preoperative PNI was 0.582, with a sensitivity of 0.272, specificity of 0.903, and Youden's index of 0.175; the AUC of the PNI on POD 1 was 0.601, with a sensitivity of 0.432, specificity of 0.785, and Youden's index of 0.217. The optimal cut-off value of the preoperative PNI for surgical complications was 40.15, while that of the PNI on POD 1 was 35.28. The results of the Delong test showed no statistical difference between the AUCs (*P* = 0.598). These data show that PNI is an important indicator for the early prediction of surgical complications and that PNI on POD 1 is more sensitive.

### Univariate and multivariate analyses of risk factors for surgical complications

After the univariate analysis on various clinical factors, the results are suggested in Table 2: including age, sex, smoking, ASA score, comorbidities, tumor location, tumor T stage, previous abdominal surgery history, neoadjuvant treatment, preoperative hemoglobin and CRP levels, preoperative lymphocyte count, surgical approach, anastomosis, stoma, operation time, intraoperative bleeding, preoperative PNI, and PNI on POD 1. Among these, male sex [odds ratio (OR) = 1.191, 95% confidence interval (CI):0.791–2.447, *P* = 0.002], other complications (respiratory, cardiovascular, and cerebrovascular system diseases) (OR = 2.295, 95% CI: 1.132–4.651, *P* = 0.021), ASA ≥3 (OR = 2.105, 95% CI: 1.102–4.023, *P* = 0.024), anastomosis (OR = 0.338, 95% CI: 0.132–0.866, *P* = 0.024), colostomy/ileostomy (OR = 2.500, 95% CI: 1.345–4.646, *P* = 0.004), preoperative PNI ≤40.15 (OR = 3.462, 95% CI: 1.656–7.238, *P* = 0.001), and PNI on POD 1 ≤35.28 (OR = 2.773, 95% CI: 1.533–5.016, *P* = 0.001) were significantly associated with surgical complications. Variables with statistical differences in the univariate analysis (*P* < 0.05) were included as covariates in the multivariate analysis model to determine the independent risk factors for surgical complications after laparoscopic surgery for rectal cancer. The association of preoperative PNI and PNI on POD 1 with surgical complications was analyzed (Table 3). Preoperative PNI ≤40.15 (OR = 2.856, 95% CI: 1.287–6.341, *P* = 0.010) and PNI on POD 1 ≤35.28 (OR = 3.118, 95% CI: 1.586–6.130, *P* = 0.001) were independent risk factors for surgical complications.

**Table 2:** Univariate analysis of risk factors associated with surgical complications.

Variable	Univariable analysis	
	OR (95% CI)	*P*
Age (>60 years)	1.191 (0.791–2.447)	0.252
Male	2.689 (1.433–5.044)	0.002
Smoking	1.300 (0.680–2.487)	0.428
Comorbidities		
Hypertension	1.520 (0.878–2.630)	0.135
Diabetes mellitus	0.924 (0.483–1.770)	0.813
Others^a^	2.295 (1.132–4.651)	0.021
ASA ≥3	2.105 (1.102–4.023)	0.024
Mid-low rectal cancer	1.521 (0.776–2.985)	0.222
Preoperative T3–4	1.643 (0.793–3.403)	0.181
Previous abdominal surgery	1.184 (0.608–2.306)	0.620
Neoadjuvant therapy	1.758 (0.987–3.133)	0.055
Preoperative hemoglobin (<120 g/L)	0.743 (0.392–1.406)	0.361
Preoperative CRP (>5 g/L)	1.086 (0.561–2.101)	0.807
Preoperative lymphocyte count ≥3 × 10^9^/L	1.787 (0.110–28.965)	0.683
Preoperative PNI ≤40.15	3.462 (1.656–7.238)	0.001
PNI on POD 1 ≤35.28	2.773 (1.533–5.016)	0.001
taTME	0.573 (0.271–1.212)	0.145
Anastomosis	0.338 (0.132–0.866)	0.024
Colostomy/ileostomy	2.500 (1.345–4.646)	0.004
Operation time >240 min	1.334 (0.718–2.479)	0.362
Blood loss >200 ml	1.346 (0.294–6.169)	0.702

ASA, American Society of Anesthesiologists; CRP, C-reactive protein; PNI, prognostic nutritional index; POD, postoperative day; taTME, transanal total mesorectal excision.

^a^ Respiratory diseases, cardiovascular and cerebrovascular diseases were included.

**Table 3:** The association between PNI and the occurrence of surgical complications.

Variable	Multivariate analysis	
	OR (95% CI)	*P*
Preoperative PNI ≤40.15^a^	2.856 (1.287–6.341)	0.010
PNI on POD 1 ≤35.28^b^	3.118 (1.586–6.130)	0.001

^a^ Model 1: Adjusted for male, other comorbidities (respiratory diseases, cardiovascular and cerebrovascular diseases), ASA ≥3, anastomosis, colostomy/ileostomy, and Preoperative PNI ≤40.15.

^b^ Model 2: Adjusted for male, other comorbidities (respiratory diseases, cardiovascular and cerebrovascular diseases), ASA ≥3, anastomosis, colostomy/ileostomy, and PNI on POD 1 ≤35.28.

### Comparison of surgical complications associated with preoperative PNI and POD 1 PNI

On the grounds of the preoperative PNI cut-off value, the patients were divided into the low (preoperative PNI ≤40.15) and high (preoperative PNI >40.15) preoperative PNI groups. They were also divided into the low (PNI on POD 1 ≤35.28) and high (PNI on POD 1 >35.28) PNI on POD 1 groups according to the PNI on POD 1 cut-off value (Table 4). Patients with a low preoperative PNI were more vulnerable to of surgical complications compared with patients with a higher one (61.1% vs. 31.2%, *P* = 0.001). In the subgroup analysis, the incidence of mild complications (Clavien–Dindo I + II) was significantly different between the two preoperative PNI groups (52.8% vs. 27.5%, *P* = 0.003). Although the incidence of major surgical complications (Clavien–Dindo III + IV) was higher in the low preoperative PNI group, the difference was not statistically significant (8.3% vs. 3.7%, *P* = 0.218). In addition, patients with preoperative PNI ≤40.15 were observed to have prolonged postoperative stay (10.4 ± 8.2 vs. 7.8 ± 3.6, *P* = 0.002). Similar trends were observed in the PNI on POD 1 group.

**Table 4:** Comparison of surgical complications in different PNI groups.

Variable	Overall (*n* = 225)	Preoperative PNI		*P*	PNI on POD 1		*P*
		≤40.15 (*n* = 36)	>40.15 (*n* = 189)		≤35.28 (*n* = 66)	>35.28 (*n* = 159)	
Overall, *n* (%)^a^^,^^b^	81 (36.0)	22 (61.1)	59 (31.2)	0.001	35 (53.0)	46 (28.9)	0.001
Grades I and II, *n* (%)^a^^,^^b^	71 (31.6)	19 (52.8)	52 (27.5)	0.003	30 (45.5)	41 (25.8)	0.004
Grade III and IV, *n* (%)^a^^,^^b^	10 (4.4)	3 (8.3)	7 (3.7)	0.218	5 (7.6)	5 (3.1)	0.142
Postoperative stay (days)^c^	10.6 ± 7.0	10.4 ± 8.2	7.8 ± 3.6	0.002	10.0 ± 7.2	7.4 ± 2.9	<0.001

PNI, prognostic nutritional index; POD, postoperative day.

^a^ Clavien–Dindo's classification of surgical complication.

^b^ Values are expressed as *n* (%).

^c^ Values are expressed as the mean ± SD.

## Discussion

The clinical significance of PNI was witnessed from the data of this study on 225 patients with rectal cancer receiving laparoscopic surgery. The results showed that low PNI values (preoperative PNI ≤40.15 and PNI on POD 1 ≤35.28) were independent risk factors for surgical complications and could lead to prolonged hospitalization. In addition, preoperative PNI and PNI on POD 1 were significantly associated with postoperative complications. However, PNI on POD 1 was more sensitive than preoperative PNI in predicting complications. Therefore, PNI is a potential and valuable predictor of surgical complications after laparoscopic surgery for rectal cancer.

PNI was originally designed to evaluate the immunonutritional status of patients undergoing surgery for gastrointestinal malignancy (6). Among the studied indicators of nutritional status, serum albumin is one of the most frequented, and lymphocyte count reflects the immune status of patients. Research on the relationship between PNI and colorectal cancer has mainly focused on the prognosis. In addition, most researches on the correlation between PNI and postoperative complications of colorectal cancer have shown that low PNI values are associated with the occurrence of severe complications; however, the relationship of PNI with minor complications has not been determined (8, 23). Rectal and colon cancers differ in the occurrence of postoperative complications, and the proportion of postoperative complications after rectal cancer surgery is higher (11). Therefore, it is of value to study the correlation between PNI and surgical complications after laparoscopic surgery for rectal cancer.

In a study on PNI in rectal cancer, Wang et al. (14) found that preoperative PNI was an independent predictive factor in predicting permanent stoma in patients who underwent AR and defunctioning stoma. Paku et al. (15) found that low preoperative PNI was a risk factor posing influence on postoperative major complications of locally recurrent rectal cancer. Xia et al. (16) found that it is rewarding to use PNI to predict postoperative complications in patients with T1–2 rectal cancer. However, these studies did not analyze overall complications after primary resection of rectal cancer and did not include postoperative PNI. Furthermore, hardly can we see studies on the correlation between PNI and surgical complications after laparoscopic surgery for rectal cancer. Evidence suggests that surgical complications of rectal cancer surgery are associated with adverse oncological outcomes (4). Therefore, early prediction and reduction of surgical complications may help to improve oncological outcomes. The correlation between PNI and surgical complications after laparoscopic surgery for rectal cancer was evaluated in this study.

The incidence of malnutrition among patients undergoing gastrointestinal surgery in hospitals is approximately 50% (24, 25). Convincing evidence has been found that nutritional risk is associated with increased complications and mortality after elective surgery (26, 27). Preoperative hypoalbuminemia is a well-known indicator of malnutrition and is viewed as a high-risk factor for postoperative complications in patients with rectal cancer (28). In addition, lymphocytes play a vital role in the host cytotoxic immune response to tumors and can be used to evaluate the health status of patients. Preoperative systemic inflammatory response through host-tumor interaction may potentially predict postoperative complications in cancer patients (29, 30). The results of this study confirm that both preoperative PNI and PNI on POD 1 are independent predictors of postoperative surgical complications after laparoscopic rectal cancer surgery. Nonetheless, PNI on POD 1 was more sensitive. On POD 1, serum samples were obtained prior to postoperative nutritional support to avoid potential bias as much as possible. Therefore, the results of the study of PNI on POD 1 are valid. Decreased postoperative serum albumin levels is closely related to systemic inflammatory response syndrome, triggering increased fractional synthesis and pathological capillary leakage of serum albumin (31). Low serum albumin level in the early postoperative period is a primary risk factor for postoperative complications after gastrointestinal surgery (32, 33). In addition, because of surgical stress, lymphocytes can change both quantitatively and qualitatively, and the number of lymphocytes gradually decreases for several days after surgery (34). This may be the reason for the higher sensitivity of the PNI on POD 1.

In this study, the preoperative PNI and PNI on POD 1 were sorted into two groups according to the cut-off value, and surgical complications were analyzed in subgroups. The results showed significant differences in the overall surgical and minor complications between the low and high PNI groups. Higher rates of major surgical complications were seen in low PNI group than the high PNI group (8.3% vs. 3.7%; 7.6% vs. 3.1%), however, no significant difference was found in the major complication subgroup (*P* = 0.218 and *P* = 0.142, respectively). The incidence of major surgical complications in this study was only 4.4% (10/225). Therefore, this may be caused by the small sample size. The predictive value of PNI for short-term major surgical complications after laparoscopic surgery for rectal cancer requires further study.

Several limitations can be found in this study. First, it was a single-center, case-control study involving just 225 patients, thus only a small sample size is available. In this case, for example, prolonged operation time increases catabolism and may induce lower levels of POD1 PNI, but no significant relationship between operation time and postoperative complications was found in this study. Second, we did not evaluate other known predictors of short-term surgical complications. Third, long-term complications were not included in this study, and further follow-up is required to obtain long-term complication results for further verification. However, the results of this study provide new ideas and possibilities for predicting and managing short-term surgical complications after laparoscopic surgery for rectal cancer.

## Conclusion

In conclusion, this study confirmed that preoperative PNI ≤40.15 and PNI on POD 1 ≤35.28 were independent risk factors for short-term surgical complications after laparoscopic rectal cancer surgery. PNI has a potential predictive value for the occurrence of short-term surgical complications after laparoscopic surgery for rectal cancer, and PNI on POD 1 was more sensitive. However, more multicenter, prospective, high-quality studies are needed to validate our results.

## Data Availability

The original contributions presented in the study are included in the article/Supplementary Material, further inquiries can be directed to the corresponding author/s.
